# Different Modulating Effects of Adenosine on Neonatal and Adult Polymorphonuclear Leukocytes

**DOI:** 10.1100/2012/387923

**Published:** 2012-08-01

**Authors:** Pei-Chen Hou, Hong-Ren Yu, Ho-Chang Kuo, Lin Wang, Li-Yan Lin, Jiunn-Ming Sheen, Te-Yao Hsu, Chia-Yu Ou, Yi-Jyun Jheng, Kuender D. Yang, Wen-Hsin Cheng

**Affiliations:** ^1^Department of Nursing, Kaohsiung Chang Gung Memorial Hospital and College of Medicine, Chang Gung University, Niao-Sung district, Kaohsiung 833, Taiwan; ^2^Department of Pediatrics, Kaohsiung Chang Gung Memorial Hospital and College of Medicine,Chang Gung University, Kaohsiung 833, Taiwan; ^3^Department of Obstetrics, Kaohsiung Chang Gung Memorial Hospital and College of Medicine, Chang Gung University, Kaohsiung 833, Taiwan; ^4^Department of Pediatrics, Show Chwan Health Care System, Changhua 505, Taiwan

## Abstract

Polymorphonuclear leukocytes (PMNs) are the major leukocytes in the circulation and play an important role in host defense. Intact PMN functions include adhesion, migration, phagocytosis, and reactive oxygen species (ROS) release. It has been known for a long time that adenosine can function as a modulator of adult PMN functions. Neonatal plasma has a higher adenosine level than that of adults; however, little is known about the modulating effects of adenosine on neonatal PMNs. The aim of this study was to investigate the effects of adenosine on neonatal PMN functions. We found that neonatal PMNs had impaired adhesion, chemotaxis, and ROS production abilities, but not phagocytosis compared to adult PMNs. As with adult PMNs, adenosine could suppress the CD11b expressions of neonatal PMNs, but had no significant suppressive effect on phagocytosis. In contrast to adult PMNs, adenosine did not significantly suppress chemotaxis and ROS production of neonatal PMNs. This may be due to impaired phagocyte reactions and a poor neonatal PMN response to adenosine. Adenosine may not be a good strategy for the treatment of neonatal sepsis because of impaired phagocyte reactions and poor response.

## 1. Introduction

Polymorphonuclear leukocytes (PMNs) are the major leukocytes in the circulation, and they play an important role in host defense. PMNs, migrating to the infection site and evoking inflammatory responses, are critical components of innate immune responses. PMNs typically use selectin receptors to form rolling attachments on the vessel wall. When stimulated, *β*2-integrin CD11b/CD18 is rapidly activated allowing leukocytes to firmly adhere to endothelial cells and migrate through the blood vessels toward sites of inflammation, following chemical signals in a process called chemotaxis [1]. PMNs are phagocytes, capable of ingesting microorganisms. They can internalize and kill many microbes, with each phagocytic event resulting in the formation of a phagosome into which reactive oxygen species (ROS) and hydrolytic enzymes are secreted.

It has been known for a long time that adenosine can function as a modulator of adult PMN functions. Four different adenosine receptors, A1, A2A, A2B, and A3, have been cloned from several species [2] and human PMNs have been found to express all four receptors [3, 4]. Adenosine regulates PMN functions with opposing effects through the A1 (immunostimulatory) and A2A (immunosuppressive) receptors. Adenosine can decrease the expression of integrins, phagocytosis, and ROS production by PMNs through A2A receptors, but enhance chemotaxis through A1 receptors [5–8]. In general, when there is significant tissue injury, the adenosine, that is generated in high concentrations by the damaged tissues, will act to inhibit PMN functions [9].

Human newborns are known to be susceptible to microbial infections. Both, innate and adaptive immunity are distinct at birth relative to adulthood. However, the mechanisms underlying the susceptibility are still incompletely defined. Neonatal plasma has a higher adenosine level than that of adults, and it contributes to a lower tumor necrosis factor (TNF) response of neonatal monocytes to lipopolysaccharides (LPS) [10]. However, little is known about the modulating effects of adenosine on neonatal PMNs. Since a better understanding of the mechanisms that control PMN functions may lead to improved treatments of inflammatory diseases, the aim of this study was to investigate the effects of adenosine on neonatal PMN functions.

## 2. Materials and Methods

### 2.1. Collection of Human Umbilical Cord Blood and Adult Peripheral Blood and Cell Separation

Human umbilical cord blood was collected into heparinized tubes (10 U/mL) by cordocentesis at the time of elective caesarean section or normal spontaneous delivery of newborns to healthy mothers, after informed consent was obtained. The peripheral blood samples were obtained from healthy adult volunteers aged 20 to 40 years. The peripheral and umbilical cord blood samples were subjected to dextran (4.5%) (Amersham Pharmacia Biotech, Uppsala, Sweden) sedimentation at a ratio of 1 : 5 to separate leukocytes from red blood cells (RBCs). The leukocytes were then separated into polymorphonuclear cells (PMNs) and mononuclear cells (MNCs) by density gradient centrifugation in Ficoll-Paque (Amersham Pharmacia Biotech) at a ratio of 2 : 1, so that there was no significant mixing of the two layers, at 1500 rpm for 30 minutes [3, 11, 12].

### 2.2. Cell Culture and Drug Treatment

Cord blood PMNs (CB PMNs) or adult peripheral blood PMNs (AB PMNs) were suspended at 1 × 10^6^ cells/mL in RPMI-1640 medium (Gibco Laboratories, Grand Island, NY) containing 10% heat-inactivated fetal bovine serum. To test the effects of exogenous adenosine on phagocyte functions, PMNs were preincubated with the indicated concentrations of adenosine (Sigma-Aldrich) for 30 min before stimulation with N-formyl-methionyl-leucyl-phenylalanine (fMLP) (Sigma Chemical Co.) or phorbol myristate acetate (PMA) (Sigma Chemical Co.).

### 2.3. Measurement of CD11b Expression

To determine the expression of CD11b, 2 × 10^6^ cells/mL were treated with the desired agents and then incubated at 37°C with or without 10^−6^ or 10^−7 ^M fMLP for up to 30 minutes. Samples were then placed on ice and stained with fluorescein- isothiocyanate- (FITC-) labeled anti-human CD11b Abs (BD Bioscience, Immunocytometry Systems, San Jose CA, USA) for 30 minutes and then washed twice by centrifugation. Negative controls were incubated with FITC-labeled mouse IgG2b (BD Bioscience) alone. Flow cytometry was performed using a FACS Calibur instrument (BD Biosciences) and analyzed using the accompanying CellQuest Pro software. Measurements are expressed as the ratio of GEOMEAN for treated PMNs compared to adenosine nontreated cells.

### 2.4. Assay of Chemotaxis

Leukocyte motility was assessed in a modified Boyden chamber as described previously [13]. Micropore membranes (5 *μ*m, Millipore Co., Bedford, MA) were layered between the upper and lower chambers. The lower chambers were filled with different concentrations of fMLP in Hanks' balanced salt solution (HBSS, Gibco-BRL, Invitrogen). PMNs (2 × 10^6^/mL) in 100 *μ*L preincubated with different concentrations of adenosine for 30 minutes were added into the upper chambers. Reactions were carried out in the presence of 5% CO_2_ at 37°C for 90 minutes. Cells that moved through the membrane completely were counted in fine random fields using a light microscope (Nikon Eclipse E400). Values are presented as the mean ± standard error based on 4 experiments, each conducted in triplicate.

### 2.5. Phagocytosis Assay

The phagocytosis assay was performed using latex bead particles for engulfment. Briefly, 200 *μ*L aliquots of neutrophils, suspended in RPMI medium (2 × 10^6^/mL) were added to the wells of a 96-well plate. Following treatment with the test compound as indicated for 30 minutes, the cells were incubated with RPMI containing 0.02% 2 *μ*m diameter FITC-latex beads (Sigma-Aldrich) and allowed to stand at 37°C for another 5, 10, 15, or 30 minutes. The cells were then washed three times and suspended in PBS. Fluorescence, derived from the FITC-latex beads ingested by the PMNs, was immediately measured using a FACScan system and CellQuest software (BD Biosciences).

### 2.6. Chemiluminescence

The assays were performed on 96-well microtiter plates, and lucigenin-enhanced chemiluminescence was read with a FluorosKan Ascent FL (Thermo Lab systems). Two hundred *μ*L of the neutrophil suspension (2 × 10^6^ cells/mL) was distributed in the wells of a 96-well microtiter plate (MaxiSorp Nunc) and incubated at different concentrations of adenosine for 30 minutes at 37°C. Thereafter, 2 *μ*L of lucigenin (5 *μ*M) and an adequate volume of the stimulus were added to the wells. PMA was added last at a final concentration of 32 *μ*M. Chemiluminescence was recorded using a Victor-3 Multilabel counter (Perkin Elmer, USA).

### 2.7. Statistics

Data are presented in relative values (%) in reference to appropriate control groups defined as 100%. All data are expressed as mean ± standard error (SE) of at least three independent experiments carried out with different blood batches and at least in duplicate. Data were analyzed by one-way ANOVA with the post hoc least significant difference test using SPSS for Windows XP (SPSS, Inc., Chicago, IL, USA). A *P* value of less than 0.05 indicated a significant difference.

## 3. Results

### 3.1. Neonatal PMNs Had a Lower CD11b Expression Than Adult PMNs upon fMLP Stimulation: Adenosine Suppressed CD11b Expression Both in Cord and Adult Blood PMNs

**Figure 1:**
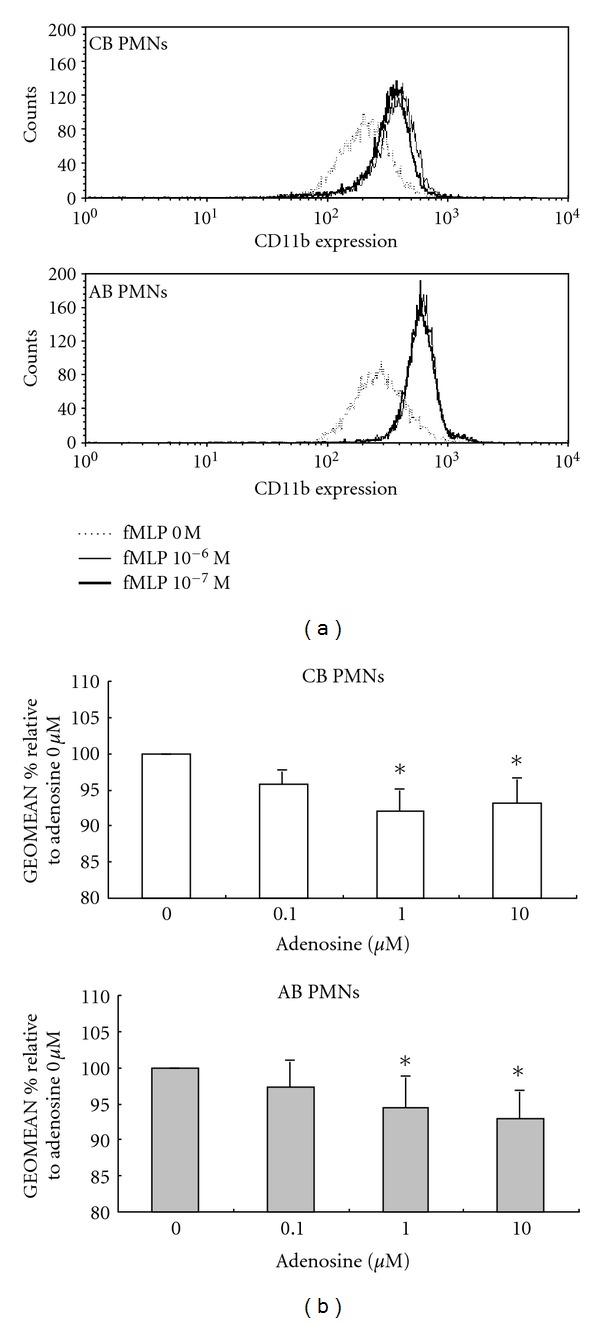
The modulating effects of adenosine on adhesion molecule expressions of neonatal and adult PMNs. (a) Cord blood PMNs always had a lower CD11b expression than adult PMNs upon fMLP stimulation. The results presented are representative of 6 duplicate experiments. (b) Plots illustrate CD11b expression levels (expressed as GEOMEAN % relative to adenosine 0 *μ*M in the ordinate) of adult and cord blood PMNs. Both in adult and cord blood PMNs, the signal intensities of CD11b were about nine percent decreased with an adenosine concentration of 1 *μ*M (compared with adenosine 0 *μ*M). Data are expressed as mean ± SD. *P < 0.05 adenosine 0 *μ*M versus adenosine 1 *μ*M and adenosine 0 *μ*M versus adenosine 10 *μ*M. Abbreviations: AB PMNs: adult peripheral blood polymorphonuclear cells; CB PMNs: cord blood polymorphonuclear cells. Data presented are calculated from 6 duplicate experiments.

CD11b is a marker of neutrophil adhesion. We first compared the CD11b expressions in CB and AB PMNs. Under resting conditions, CB and AB PMNs had similar CD11b expressions. With fMLP stimulation, the CD11b expressions were increased both in CB and AB PMNs (Figure 1(a)). fMLP concentrations of 10^−6^ and 10^−7 ^M had the same stimulating effects on CD11b expression. However, AB PMNs always had a higher CD11b expression than CB PMNs upon fMLP stimulation (Figure 1(a)). In order to study the modulating effect of adenosine on CD11b, AB and CB PMNs were stimulated with fMLP (10^−7 ^M) after incubation in medium containing different concentrations of adenosine for 30 minutes. The signal intensities of CD11b decreased when the adenosine concentration was above 1 *μ*M (Figure 1(b)).

### 3.2. Neonatal PMNs Had Lower Chemotactic Ability Than Adult PMNs: Adenosine Suppressed Chemotaxis of Adult Blood PMNs

**Figure 2:**
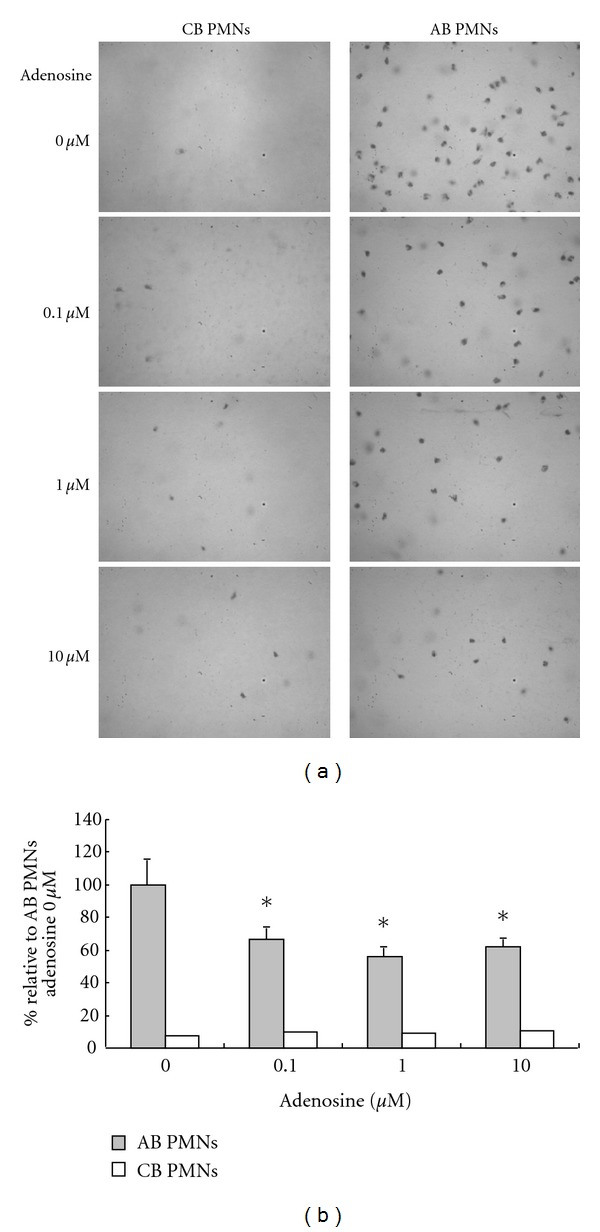
The modulating effects of adenosine on chemotaxis of neonatal and adult PMNs. (a) Cord blood PMNs always had a lower chemotactic ability than adult PMNs upon fMLP stimulation. The results are representative of 6 duplicate experiments. (b) Plots illustrate chemotactic ability (expressed as numeral of number relative to adenosine 0 *μ*M in the ordinate) of adult and cord blood PMNs. The chemotactic ability of adult PMNs was decreased by about 40% at an adenosine concentration of 0.1 *μ*M (compared with adenosine 0 *μ*M). Data are expressed as mean ± SE. Abbreviations: AB PMNs: adult peripheral blood polymorphonuclear cells; CB PMNs: cord blood polymorphonuclear cells. Data presented are calculated from 6 duplicate experiments. *P < 0.05 as compared with adenosine 0 *μ*M.

Another important function of PMNs is chemotaxis. We, therefore, evaluated the effect of adenosine on AB and CB PMNs. AB or CB PMNs were incubated in medium containing the indicated concentrations of adenosine for 30 minutes, and their chemotactic abilities were tested with fMLP stimulation. As showed in Figure 2(a), CB PMNs had a greater impairment of chemotactic ability compared to AB PMNs. As the adenosine concentration increased, AB PMNs showed depressed chemotactic expressions in a dose dependent manner (Figures 2(a) and 2(b)). However, the effect of adenosine on CB PMN chemotaxis was not obvious.

### 3.3. The Phagocytic Ability of Neonatal PMNs Was Comparative with Adult PMNs: Adenosine Has No Obvious Modulation Effect on Phagocytosis

**Figure 3:**
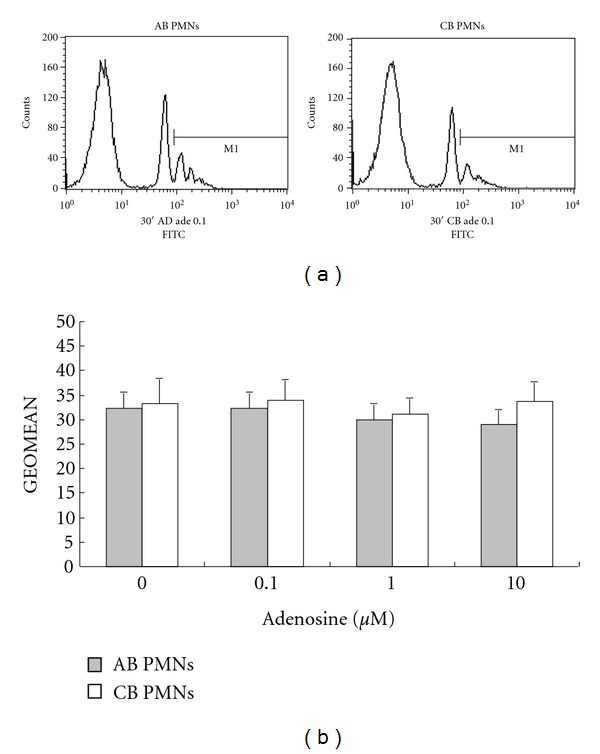
The modulating effects of adenosine on phagocytosis of neonatal and adult PMNs. (a) The phagocytotic manifestation of cord blood PMNs was comparable with adult PMNs. The results are representative of 6 duplicate experiments. (b) Plots illustrate phagocytosis (expressed as GEOMEAN in the ordinate) of adult and cord blood PMNs. Neither adult nor cord blood PMNs phagocytosis was influenced by adenosine (Figures 3(a) and 3(b)).

To identify whether adenosine could modulate the phagocytotic ability of AB and CB PMNs, AB or CB PMNs were first incubated at the indicated concentrations of adenosine for 30 minutes, and then cocultured with beads coated with fluorescent dye. The phagocytotic ability of the AB and CB PMNs was analysed using fluorocytometry and found to be comparable. Neither AB nor CB PMN phagocytosis was influenced by adenosine (Figures 3(a) and 3(b)).

### 3.4. Neonatal PMNs Had Lower Oxygen Radical Release Than Adult PMNs: Adenosine Delayed Oxygen Radical Release of Adult PMNs

**Figure 4:**
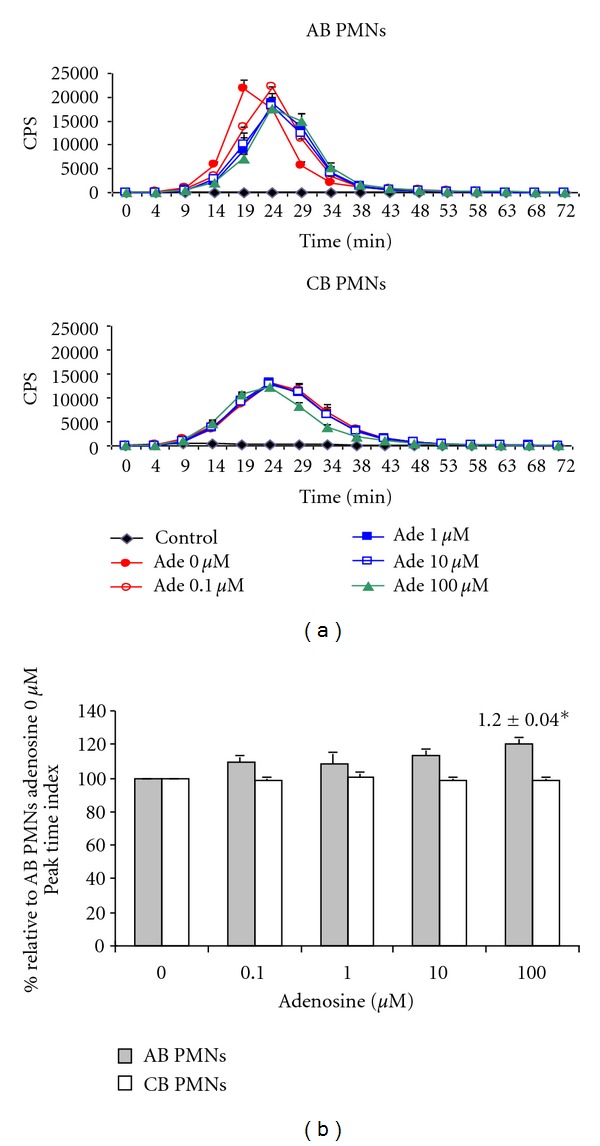
The modulating effects of adenosine on oxygen radical release of neonatal and adult PMNs. (a) The amount of oxygen free radicals released by PMNs was determined by chemiluminescence. Cord blood PMNs had lower PMA-induced oxygen free radical production than adult PMNs. The results are representative of 6 duplicate experiments. (b) Plots illustrate the relative peak time of oxygen radicals released by adult and cord blood PMNs. The results were normalized to those obtained in pair-controlled studies of adult PMNs with adenosine 0 *μ*M. Data are expressed as mean ± SD. *P < 0.05 as compared with adenosine 0 *μ*M. Data presented are calculated from 6 duplicate experiments.

To explore the effects of adenosine on oxygen free radical released by PMNs, AB and CB PMNs were first incubated at the indicated concentrations of adenosine for 30 minutes, and then stimulated with PMA. The amount of oxygen free radicals released by the PMNs was determined by chemiluminescence. The PMA-induced oxygen free radical production activity of the neonatal PMNs was significantly lower than that of the adult group (1.84 ± 0.87 × 10^7^ versus 4.20 ± 0.77 × 10^7^ cps; *P* < 0.05). We found that oxygen free radical production by the AB PMNs, but not the CB PMNs, was delayed as the adenosine level increased (Figures 4(a) and 4(b)). However, the total amount of oxygen free radicals released, by the AB or CB PMNs, was not influenced by adenosine (data not shown).

## 4. Discussion

In this study, we evaluated the effects of adenosine on neonatal PMN functions. Our findings are in agreement with previous reports that neonatal PMNs are impaired in adhesion, chemotaxis, and ROS production abilities, but not phagocytosis compared to adult PMNs [14–18]. This partly contributes to the susceptibility of newborns to bacterial infections.

Adenosine can suppress the generation of superoxide and inhibit adhesion and recruitment of adult PMNs. Neonatal plasma has a higher level of adenosine than adult plasma, and this accounts for reduced BLP-induced-monocyte synthesis of TNF-*α* [10]. A lower amount of adenosine deaminase in cord blood mononuclear cells than in adult peripheral blood mononuclear cells partly explains the higher adenosine levels in neonatal plasma [19]. Neonatal monocytes have also been reported to have heightened sensitivity to adenosine A3 receptor-mediated actions [10]. However, little is known about the modulating effects of adenosine on neonatal PMN functions. We found that, as in adult PMNs, adenosine suppressed the CD11b expression of neonatal PMNs, but with no significant suppressive effect on phagocytosis. In contrast to adult PMNs, adenosine did not significantly suppress chemotaxis and ROS production of neonatal PMNs. This may be due to impaired phagocyte reactions and a poor response to adenosine of neonatal PMNs.

 Adenosine A1 receptors were stimulated by 10^−4^ to 10^−2^ *μ*M concentrations of adenosine and lead to a decrease in intracellular cAMP levels. In contrast, adenosine A1 receptors have been found to be stimulated by 10^−4^ to 10^−2^ *μ*M concentrations of adenosine, leading to a decrease in intracellular cAMP levels. In contrast, adenosine A2A receptors and A2B receptors have been found to be stimulated by higher (5 × 10^−1^ *μ*M and 10 *μ*M, resp.) concentrations of adenosine, leading to an increase in cAMP levels [20]. Different adenosine receptors mediate even conflicted responses. Thus, the function of adenosine is determined partly by its concentration. Adenosine is constitutively present in the extracellular space at low levels (<1 *μ*M). However, its concentration may increase by tenfold under certain inflammatory conditions that exert an immunosuppressive effect [21]. Although several adenosine receptor subtype agonists are available, to more closely resemble physiological conditions, we choose adenosine as the experimental reagent for this study.

 Adenosine A2A receptors have been shown to exert potent anti-inflammatory and immunosuppressive effects. Recently, a study demonstrated that adenosine allowed for the selective suppression of tissue-toxic oxygen reactive metabolite production via the engagement of A2A receptor sites, without compromising microbicidal effector functions of PMNs. Thus, infusion of adenosine may provide a new perspective in the treatment of sepsis. Neonates are particularly susceptible to sepsis compared to older children and adults (5.3/1,000 versus 0.2/1,000) and are then prone to morbidity and mortality [22, 23]. However, due to the impaired immune reactions and poor response to adenosine of neonatal PMNs, treatment with adenosine does not seem to be a good strategy for neonatal sepsis.

 In conclusion, the results of this study demonstrate that neonatal PMNs have impaired adhesion, chemotaxis, and ROS production abilities, but not phagocytosis compared to adult PMNs. A higher concentration of adenosine in neonates contributes to a decrease in adhesion molecule expression of neonatal PMNs. Adenosine may not be a good strategy for the treatment of neonatal sepsis because of impaired phagocyte reactions and a poor response to adenosine.
